# The opposing trends of body mass index and blood pressure during 1977–2020; nationwide registry of 2.8 million male and female adolescents

**DOI:** 10.1186/s12933-021-01433-0

**Published:** 2021-12-28

**Authors:** Boris Fishman, Yair Zloof, Omri Orr, Avishai M. Tsur, Ariel Furer, Ma’ayan Omer Gilon, Gabriel Chodick, Adi Leiba, Estela Derazne, Dorit Tzur, Arnon Afek, Ehud Grossman, Gilad Twig

**Affiliations:** 1 Department of Military Medicine, Hebrew University, Jerusalem and the Israel Defense Forces Medical Corps, Ramat Gan, Israel; 2 Division of Cardiology, The Leviev Heart Center, The Chaim Sheba Medical Center, Tel Hashomer, Ramat Gan, Israel; 3 Sackler Faculty of Medicine, Tel Aviv University, Tel Aviv, Israel; 4 The Talpiot Sheba Medical Leadership Program, Ramat Gan, Israel; 5 Orthopedic Surgery, Rambam Health Care Campus, Haifa, Israel; 6 Department of Medicine ‘B’, Zabludowicz Center for Autoimmune Diseases, Sheba Medical Center, Tel-Hashomer, Ramat Gan, Israel; 7 Maccabitech, Maccabi Healthcare Services, Tel Aviv, Israel; 8 Division of Nephrology and Hypertension, Assuta Ashdod Academic Medical Center affiliated to Ben Gurion University, Beer Sheva, Israel; 9 Harvard Medical School, Boston, MA USA; 10 Central Management, The Chaim Sheba Medical Center, Tel Hashomer, Ramat Gan, Israel; 11 The Hypertension Unit and the Internal Division, The Chaim Sheba Medical Center, Tel Hashomer, Ramat Gan, Israel; 12 Institute of Endocrinology, Sheba Medical Center, Tel Hashomer, Ramat Gan, Israel

**Keywords:** Systolic blood pressure, Diastolic blood pressure, Hypertension, Body Mass Index, Obesity, Adolescents

## Abstract

**Background:**

Elevated blood pressure among adolescents has been shown to be associated with future adverse cardiovascular outcomes and early onset diabetes. Most data regarding systolic and diastolic blood pressure trends are based on surveys of selected populations within 10–20-year periods. The goal of this study was to characterize the secular trend of blood pressure given the rising prevalence of adolescent obesity.

**Methods:**

This nationwide population-based study included 2,785,515 Israeli adolescents (41.6% females, mean age 17.4 years) who were medically evaluated and whose weight, height and blood pressure were measured, prior to mandatory military service between 1977 and 2020. The study period was divided into 5-year intervals. Linear regression models were used to describe the P for trend along the time intervals. Analysis of covariance was used to calculate means of blood pressure adjusted for body mass index.

**Results:**

During the study period, the mean body mass index increased by 2.1 and 1.6 kg/m^2^ in males and females, respectively (P for trend  < 0.001 in both sexes). The mean diastolic blood pressure decreased by 3.6 mmHg in males and by 2.9 mmHg in females (P  < 0.001 in both sexes). The mean systolic blood pressure increased by 1.6 mmHg in males and decreased by 1.9 mmHg in females. These trends were also consistent when blood pressure values were adjusted to body mass index.

**Conclusion:**

Despite the increase in body mass index over the last four decades, diastolic blood pressure decreased in both sexes while systolic blood pressure increased slightly in males and decreased in females.

**Supplementary Information:**

The online version contains supplementary material available at 10.1186/s12933-021-01433-0.

## Background

Elevated blood pressure (BP) among the general population is a leading contributor to cardiovascular morbidity and mortality [1, 2]. A number of studies have reported associations of adolescent elevated BP with adult hypertension and with renal and cardiovascular morbidity and mortality later in life [3–6]. Furthermore, adolescent elevated BP was found to promote early onset type 2 diabetes mellitus [5].

Weight status is a key determinant of BP in adolescents [7]. Consistent increases have been shown in childhood and adolescent obesity in most Western countries [8, 9], including Israel and the US, where adolescent obesity has nearly tripled over the last decades [10, 11]. Most relevant studies in Western countries reported decreases over the years in diastolic BP (DBP), and to a lesser extent, in systolic BP (SBP) [12–18]. Those studies were based on relatively small samples of wide age range, including both pediatric and adolescent populations. The aim of this study was to describe secular trends during 44 years, of measured SBP and DBP, while accounting for the trend in body mass index (BMI), among 2.78 million adolescents screened at age 17 years.

## Methods

### Databases and study population

**Fig. 1:**
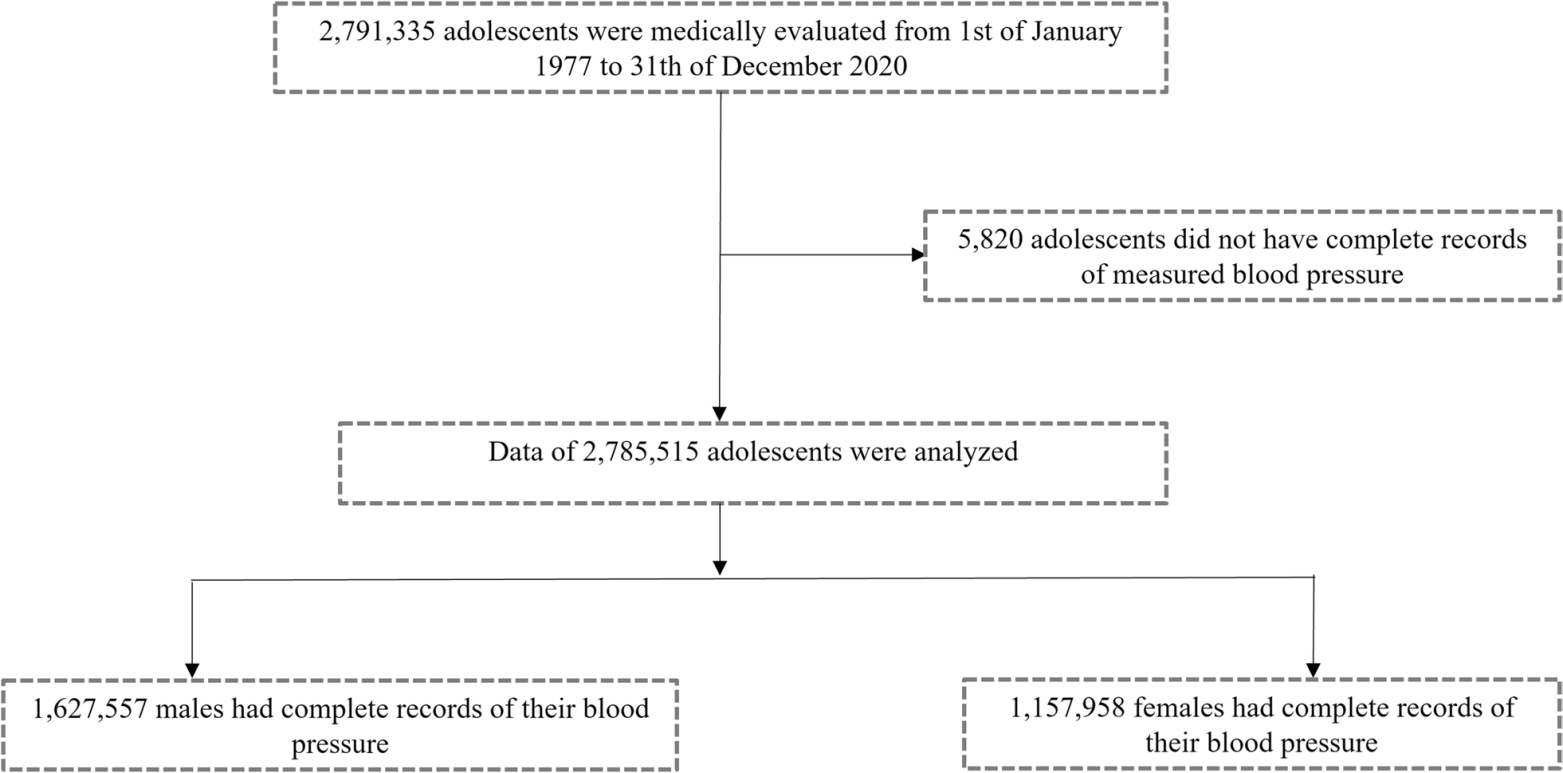
Study population

One year prior to mandatory military service, usually at age 16–19 years, male and female Israeli adolescents are called to regional conscription centers for medical evaluation. The current study comprised adolescents examined during 1977–2020, regardless of their fitness for military service. Orthodox and ultra-orthodox Jewish women are not obligated to serve, and therefore are under-represented [19]. We excluded adolescents with missing values of BP or BMI data (0.2% in total). Figure 1 presents the study design. The Institutional Review Board of the Israel Defense Forces Medical Corps approved the study.

### Data collection at regional military recruitment centers

The medical assessment included review of the examinee’s medical history, an interview with a physician, physical examination and further medical tests as appropriate [20, 21]. Height and weight were measured (barefoot and in underwear) by trained medics, and BMI was calculated (weight in kilograms divided by height squared in meters) [22]. We used the BMI classification of the US Centers for Disease Control and Prevention, which was validated and selected by the Israeli Ministry of Health as the routine reference for anthropometric data for Israeli children and adolescents [23]. Separately for males and females [24], the study population was divided into four BMI-for-age percentile groups [25]: underweight (< 5th percentile), normal weight (5–84th), overweight (85–94th) and obese (≥ 95th). SBP and DBP were routinely recorded, based on the measurement on the right arm acquired while sitting quietly for at least 5 min. Until 2005, manual sphygmomanometers were used (1977–1985, mercury sphygmomanometers; 1986–2005, aneroid sphygmomanometers). From 2006, digital oscillometric devices gradually replaced manual ones.

Data regarding years of schooling were obtained from the Ministry of Education and were dichotomized at 11 years (< 11/≥ 11 years) [10]. Data regarding immigration history and place of residence were received from the Ministry of Interior [26]. Residential socioeconomic status was classified into three groups (low/medium/high) based on criteria of the Israeli Bureau of Statistics [26, 27].

### Statistical analysis

We a priori stratified analyses by sex and divided the study period into 5-year time intervals (1977–1980, 1981–1985, 1986–1990, 1991–1995, 1996–2000, 2001–2005, 2006–2010, 2011–2015 and 2016–2020). We calculated the P for trend for all the study variables including SBP, DBP and BMI using linear regression models. We plotted mean SBP, DBP and BMI values for the time intervals.

Two analytical approaches were taken to address the potential effect of the rise in adolescent BMI throughout the study period [10], in light of its tight relationship with BP levels [10]. (i) Mean SBP and DBP values were adjusted for BMI in each time interval using analysis of covariance (ANCOVA). We calculated the regression coefficients of the trends in SBP and DBP in unadjusted (β_unadjusted_) and BMI-adjusted (β_BMI-adjusted_) models, which reflected the predicted mean change in mmHg per 5-year interval. (ii) We stratified the secular trend of mean SBP and DBP by BMI categories (underweight/normal BMI/overweight/obese). Additional sensitivity analyses included the following: (1) limiting the study sample to persons with unimpaired health (apart from hypertension, a lack of chronic medical treatment, or a history of major surgery or cancer) in order to minimize confounding by coexisting comorbidities. (2) We included only Israeli-born adolescents to reduce health effects related to immigration [26]. The IBM SPSS software program version 25 was used for statistical analysis.

## Results

The final study population comprised 2,785,515 adolescents; 1,157,958 (41.6%) were females. The characteristics at assessment are shown separately for males and females in Tables 1, 2, respectively. The overall mean age at assessment was 17.4 (SD; 0.6) years in both sexes. During the study period, the mean height increased by approximately 1 cm in males, and remained stable in females. There was a trend of increasing mean BMI: from 20.7 (SD; 2.8) kg/m^2^ for males and 21.0 (2.9) kg/m^2^ for females in the earliest time interval, to 22.8 (4.4) kg/m^2^ and 22.6 (4.4) kg/m^2^, respectively (P for trend  < 0.001 in both sexes; Tables 1, 2). This increase was accompanied by increased prevalences of adolescent overweight and obesity (P for trend  < 0.001 in both sexes; Tables 1, 2).

**Table 1:** Characteristics of males by time intervals (n  = 1,627,557)

	1977–1980 (n = 97,483)	1981–1985 (n = 1,35,678)	1986–1990 (n = 1,57,008)	1991–1995 (n = 2,05,252)	1996–2000 (n = 2,07,875)	2001–2005 (n = 2,05,692)	2006–2010 (n = 1,93,699)	2011–2015 (n = 2,18,790)	2016–2020 (n = 2,06,080)	Total (n = 16,27,557)	P for trend
Age (mean ± SD)	17.4 ± 0.5	17.4 ± 0.5	17.4 ± 0.5	17.5 ± 0.8	17.5 ± 0.8	17.3 ± 0.6	17.3 ± 0.6	17.4 ± 0.8	17.4 ± 0.7	17.4 ± 0.7	0.12
Height (cm ± SD)	172.9 ± 6.6	173.3 ± 6.7	173.9 ± 6.7	174.3 ± 6.8	174.2 ± 6.9	173.9 ± 6.8	174.2 ± 6.7	173.8 ± 6.8	174.2 ± 6.8	174 ± 6.8	0.046
BMI (mean ± SD)	20.7 ± 2.8	20.6 ± 2.9	21.1 ± 3.1	21.1 ± 3.3	21.1 ± 3.5	21.6 ± 3.8	21.9 ± 4.0	22.4 ± 4.35	22.8 ± 4.4	21.5 ± 3.7	< 0.001
CDC BMI groups (% of each BMI percentile category)											< 0.001
Underweight	8058 (8.3)	12,513 (9.3)	13,828 (8.9)	17,000 (8.6)	18,334 (9.1)	15,578 (7.7)	13,081 (6.8)	13,472 (6.3)	13,556 (6.7)	1,24,420 (7.8)	
Normal weight	81,228 (84.2)	1,11,594 (82.9)	1,25,690 (80.7)	1,57,596 (79.5)	1,57,312 (77.8)	1,53,480 (75.9)	1,41,329 (73.8)	1,52,355 (70.9)	1,42,770 (70.7)	12,23,354 (76.6)	
Overweight	5326 (5.4)	7429 (5.5)	11,223 (7.2)	15,606 (7.9)	16,508 (8.2)	19,816 (9.8)	21,381 (11.2)	27,084 (12.6)	24,796 (12.3)	1,49,079 (9.3)	
Obese	1986 (2.1)	3050 (2.3)	5030 (3.2)	7950 (4.0)	10,052 (5.0)	13,375 (6.6)	15,893 (8.2)	22,114 (10.3)	20,776 (10.3)	1,00,026 (6.3)	
Immigrants (% of time interval)	18,842 (19.3)	15,576 (11.5)	13,872 (8.8)	37,962 (18.5)	47,970 (23.1)	46,705 (22.7)	34,773 (18.0)	24,944 (11.4)	19,012 (9.2)	2,59,656 (16.0)	0.97
Years of education ≥ 11 (% of time interval)	61,361 (63.2)	99,202 (73.4)	12,968 (82.8)	1,85,590 (90.6)	1,91,457 (92.1)	1,93,326 (94.0)	1,81,861 (94.0)	2,00,850 (92.1)	1,73,019 (94.5)	1,416,346 (88.4)	0.004
Socioeconomic status (% of each category)											0.37
Low	29,456 (30.7)	41,462 (30.9)	45,283 (29.1)	58,851 (29.0)	58,187 (28.1)	58,214 (28.4)	57,367 (29.7)	71,381 (32.8)	58,985 (28.9)	4,79,195 (29.7)	
Medium	45,714 (47.6)	64,026 (47.7)	76,167 (49.0)	1,02,573 (50.5)	1,09,380 (52.9)	1,06,996 (52.2)	98,301 (51.0)	1,04,099 (47.9)	1,01,908 (50.0)	8,09,164 (50.1)	
High	20,807 (21.7)	28,628 (21.3)	34,051 (21.9)	41,798 (20.6)	39,138 (18.9)	39,689 (19.4)	37,255 (19.3)	41,987 (19.3)	43,061 (21.1)	3,26,414 (20.2)	

*CDC* US Center of Disease Control; *BMI* body mass index (calculated as weight in kilograms divided by height in meters squared); *SD* standard deviations; *cm* centimeters; *kg* kilograms

**Table 2:** Characteristics of females by time intervals (n  = 1,157,958)

	1977–1980 (n = 58,181)	1981–1985 (n = 90,198)	1986–1990 (n = 1,09,192)	1991–1995 (n = 1,43,745)	1996–2000 (n = 1,50,368)	2001–2005 (n = 1,53,276)	2006–2010 (n = 1,45,457)	2011–2015 (n = 1,53,623)	2016–2020 (n = 1,53,908)	Total (n = 11,57,958)	P value for trend
Age (mean ± SD)	17.5 ± 0.4	17.4 ± 0.4	17.4 ± 0.3	17.3 ± 0.3	17.3 ± 0.4	17.2 ± 0.4	17.1 ± 0.4	17 ± 0.6	17.0 ± 0.5	17.2 ± 2.7	0.48
Height (cm ± SD)	161.9 ± 5.9	162.1 ± 5.9	162.5 ± 6.0	162.1 ± 6.2	162.0 ± 6.2	162.1 ± 6.3	161.8 ± 6.2	162.2 ± 6.3	162.2 ± 6.2	162.2 ± 6.2	0.51
BMI (mean ± SD)	21.0 ± 2.9	20.9 ± 3.0	21.1 ± 3.2	21.1 ± 3.4	21.2 ± 3.5	21.2 ± 3.7	21.5 ± 3.9	22 ± 4.2	22.6 ± 4.3	21.5 ± 3.7	0.002
CDC BMI groups (% of each BMI percentile category)											0.001
Underweight	2229 (3.8)	3927 (4.4)	4581 (4.2)	6741 (4.7)	7127 (4.7)	7502 (4.9)	7045 (4.9)	6213 (4.1)	5806 (3.8)	51,171 (4.4)	
Normal weight	50,633 (87.3)	77,811 (86.5)	92,536 (84.9)	1,19,379 (83.1)	1,23,537 (82.3)	1,23,963 (81.1)	1,14,648 (79.0)	1,17,181 (76.8)	1,14,940 (75.2)	9,34,628 (81.0)	
Overweight	4438 (7.7)	6917 (7.7)	9590 (8.8)	13,536 (9.4)	14,506 (9.7)	15,387 (10.1)	16,294 (11.2)	19,879 (13.0)	21,142 (13.8)	1,21,689 (10.5)	
Obese	704 (1.2)	1249 (1.4)	2327 (2.1)	3934 (2.7)	4891 (3.3)	6051 (4.0)	7049 (4.9)	9377 (6.1)	10,918 (7.1)	46,500 (4.0)	
Immigrants (% of time interval)	8548 (14.7)	8206 (9.1)	7327 (6.7)	16,307 (11.3)	27,728 (18.4)	33,836 (221)	26,814 (18.4)	18,207 (11.9)	14,063 (9.1)	1,61,036 (13.9)	0.61
Years of education ≥ 11 (% of time interval)	48,412 (85.3)	80,902 (91.0)	1,04,099 (96.6)	1,39,508 (97.6)	1,45,783 (97.0)	1,49,065 (97.3)	1,42,693 (98.1)	1,50,279 (98.1)	1,33,567 (97.8)	1,094,308 (96.4)	0.02
Socioeconomic status (% of each category)											0.19
Low	11,500 (20.7)	19,582 (22.2)	21,518 (20.1)	28,739 (20.2)	33,465 (22.3)	33,790 (22.1)	32,358 (22.3)	32,031 (20.9)	31,633 (20.7)	2,44,616 (21.3)	
Medium	28,350 (51.1)	45,566 (51.6)	58,008 (54.1)	78,144 (55.0)	82,946 (55.3)	85,663 (56.0)	79,580 (54.8)	83,523 (54.6)	83,251 (54.4)	6,25,031 (54.5)	
High	15,622 (28.2)	23,099 (26.2)	27,757 (25.9)	35,140 (24.7)	33,483 (22.3)	33,524 (21.9)	33,350 (23.0)	37,504 (24.5)	38,052 (24.9)	2,77,531 (24.2)	

*CDC* US Center of Disease Control; *BMI* body mass index (calculated as weight in kilograms divided by height in meters squared); *SD* standard deviations; *cm* centimeters; *kg* kilograms

**Fig. 2:**
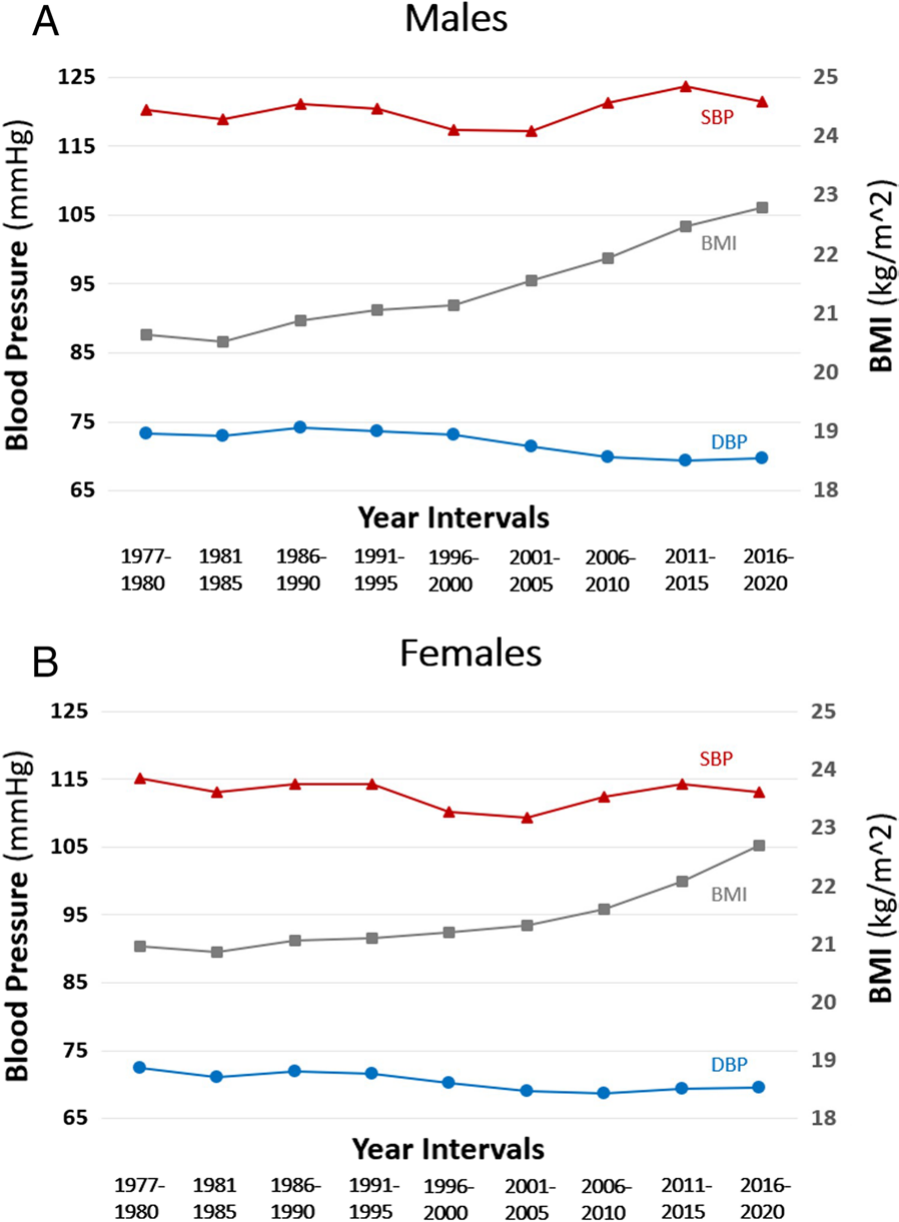
The secular trend of systolic blood pressure, diastolic blood pressure and BMI among **A** males (n  = 1,627,557) and **B** females (n  = 1,157,958) over the study period (1977–2020), by 5-year time intervals. *BMI* body mass index (weight in kilograms divided by height squared in meters); *SBP* systolic blood pressure (mm/Hg); *DBP* diastolic blood pressure; *SD* standard deviation

Mean DBP decreased in both sexes during the study period (P for trend  < 0.001; Table 3; Fig. 2). The overall mean decrease was 3.6 mmHg (β_unadjusted_ = − 0.65 mmHg/5-year, β_BMI-adjusted_  = − 0.74 mmHg/5-year) in males and 2.9 mmHg (β_unadjusted_  = − 0.41 mmHg/5-year, β_BMI-adjusted_  = − 0.48 mmHg/5-year) in females. The SBP trend was not consistent between the sexes. In males, we recorded a small increase in mean SBP during the study period, of 1.6 mmHg (P for trend  < 0.001; (β_unadjusted_  = 0.39 mmHg/5-year, β_BMI-adjusted_  = 0.17 mmHg/5-year). In females we observed a decrease of 1.9 mmHg (P for trend  < 0.001 β_unadjusted_  = − 0.10, β_BMI-adjusted_  = − 0.25).

**Table 3:** Recorded BMI, crude systolic and diastolic blood pressure values, and BMI adjusted levels, separately for males (a) and females (b)

Years	BMI		Recorded SBP		BMI adjusted SBP	Recorded DBP		BMI adjusted DBP
	Mean	SD	Mean	SD	Mean	Mean	SD	Mean
A. Males (n = 1,627,557)								
1977–1980	20.66	2.83	120.00	12.19	120.90	73.29	8.10	73.67
1981–1985	20.57	2.88	118.91	11.94	119.87	72.97	8.02	73.43
1986–1990	20.91	3.13	121.13	11.95	121.66	74.12	8.17	74.37
1991–1995	21.13	3.29	120.43	11.69	120.76	73.76	7.99	73.92
1996–2000	21.18	3.50	117.48	11.11	117.74	73.29	7.96	73.42
2001–2005	21.55	3.77	117.37	11.15	117.39	71.62	7.90	71.63
2006–2010	21.92	4.05	121.56	11.87	121.31	70.03	8.48	69.93
2011–2015	22.45	4.35	123.85	12.29	123.23	69.51	9.00	69.25
2016–2020	22.78	4.41	121.60	12.09	120.80	69.73	8.86	69.38
B. Females (n = 1,157,958)								
1977–1980	20.99	2.91	115.12	11.7	115.49	72.49	8.03	72.70
1981–1985	20.88	2.98	113.07	11.49	113.53	71.12	8.00	71.38
1986–1990	21.1	3.18	114.36	11.89	114.62	71.99	8.37	72.14
1991–1995	21.14	3.36	114.25	12.09	114.48	71.68	7.94	71.81
1996–2000	21.19	3.50	110.38	11.31	110.58	70.29	8.02	70.40
2001–2005	21.25	3.70	109.46	11.02	109.62	69.31	7.95	69.40
2006–2010	21.53	3.92	112.60	11.79	112.57	68.98	8.23	69.97
2011–2015	22.04	4.18	114.51	11.94	114.11	69.47	8.55	69.28
2016–2020	22.6	4.35	113.21	11.32	112.41	69.60	8.39	69.26

The recorded blood pressure values were retrieved directly from the Israeli Defense Forces Registry. BMI adjusted values of SBP and DBP were calculated by analysis of covariance (ANCOVA) using a general linear model

*BMI* body mass index (weight in kilograms divided by height squared in meters); *SBP* systolic blood pressure (mm/Hg); *DBP* diastolic blood pressure; *SD* standard deviation

**Fig. 3:**
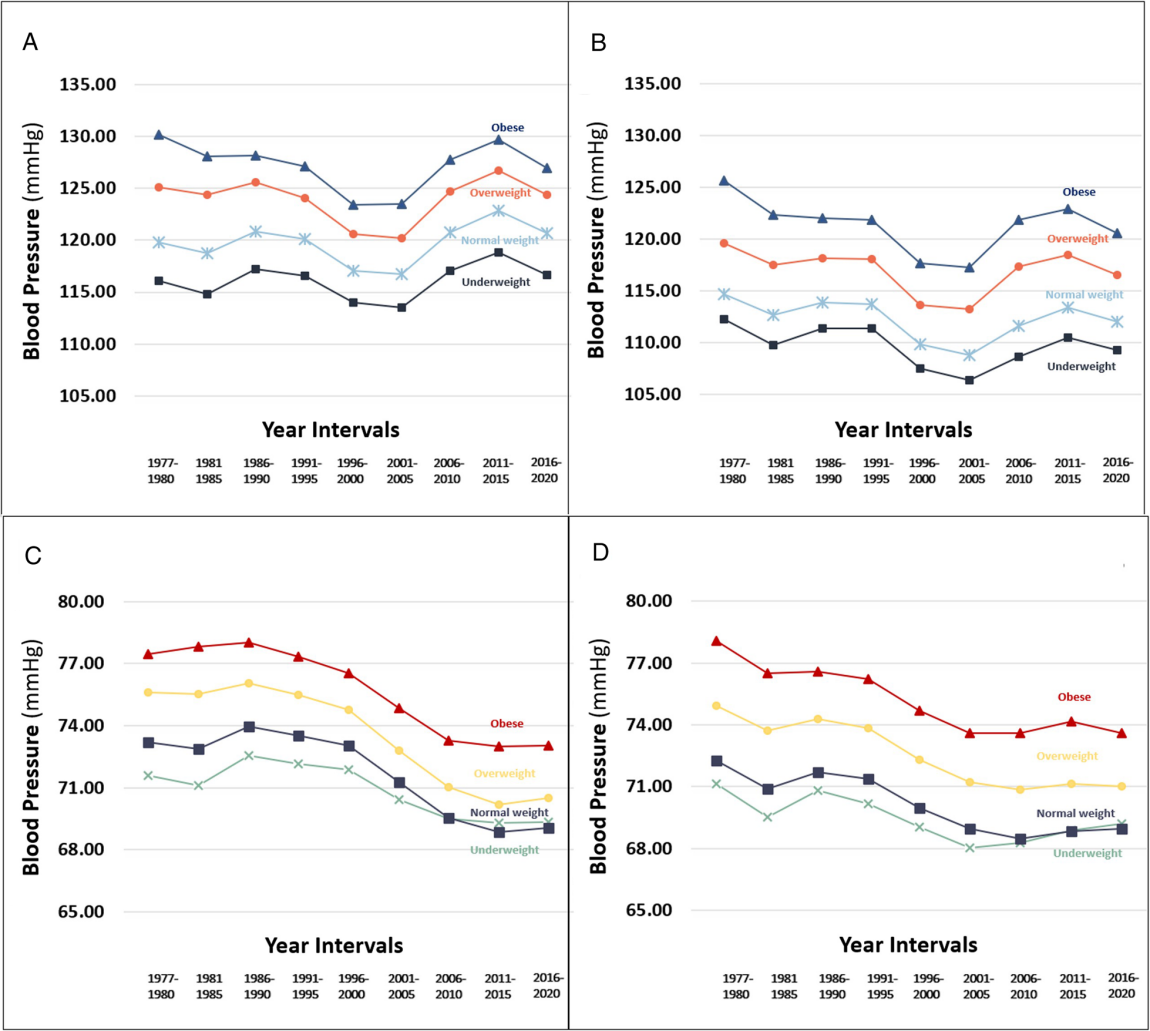
The secular trends of systolic blood pressure and diastolic blood pressure by BMI group, separately for males (**A**, **C**) and females (**B**, **D**) by 5-year time intervals. The BMI groups are defined by the US Center of Disease Control: underweight (< 5th percentile), normal weight (5–84th), overweight (85–94th) and obese (≥ 95th)

The trends of SBP and DBP levels remained stable throughout the study period, across all BMI categories (Fig. 3). For both sexes, mean SBP and DBP values in every time interval were highest in the obese group, followed by the overweight group, compared to the other BMI groups. The secular trends of BMI-adjusted DBP (Additional file 1: Figure S1) followed trends of the crude BP values in both sexes (P for trend for both sexes  < 0.001, Table 3). The results persisted when the study population was limited to those with unimpaired health (Additional file 1: Figure S2), and to those born in Israel only (Additional file 1: Figure S3).

## Discussion

In this nationwide study we demonstrated a trend of a slight increase in SBP, of 1.6 mmHg, among males; and a small decrease, of 1.9 mmHg, among females, over the course of four decades. Meanwhile, DBP exhibited mean decreases of 3.6 and 2.9 mmHg among males and females, respectively. These trends remained after accounting for increases in BMI throughout the study period, and in all strata of BMI categories.

A pooled analysis of 19 million adults in Western countries showed a trend of decreasing SBP and DBP [28]. Among the adolescent population, several studies from the US reported minimal decreases in SBP, of up to 0.8 mm/Hg, during 13–20 years (1974–1994 [14], 1999–2012 [17] and 1998–2018 [12]). Reports from other Western countries such as Northern Ireland (during the years 1999–2009) [18], Japan (1994–2010) [15] and Korea (1998–2008) [29] reported much larger SBP decrements, of 7–10 mmHg. DBP decrements of 2–4 mmHg were reported among American and Korean adolescents [12, 14, 17, 29], and up to 11 mmHg in the Northern Ireland population [18]. Our results concur with most reports that were based on surveys originated from the United States.

For a time interval of 44 years, we report overall increases in mean BMI of 2.1 and 1.6 kg/m^2^ among male and female adolescents, respectively. This concurs with studies that reported increases over recent decades in BMI in developed countries; and following the industrial process, in developing countries as well. Globally, mean BMI increases were estimated as about 0.5 kg/m^2^ per decade [30, 31]. As expected, throughout the period of the current study, mean BP values were significantly higher among those with overweight and obesity than normal weight, emphasizing the effect of BMI on BP [32, 33]. Yet, the trends of SBP and DBP were consistent regardless of BMI category, and most BMI-adjusted analyses demonstrated similar trends to those of the crude BP values. Therefore, it is unlikely that BMI solely accounts for the BP trends described in this study. Notably, the impact of increased BMI (overweight and obesity compared to normal weight) in regard to SBP and DBP was more pronounced in females than in males, despite a similar increase in the proportion of overweight and obesity in both sexes during the study period. This observation may be explained by the occurrence of increased BMI that represents increased muscle among a higher proportion of males than females [34, 35].

Alternatively, our results may stem from changes in demographic composition, mostly due to immigration [36]. Starting from the 1990s and continuing until 2010, the proportion of immigrants from the entire examinees’ population increased to 23% (compared to approximately 10% in the 1980s). This mostly comprised Ashkenazi Jews immigrating from the countries of the former Union of Soviet Socialist Republics. Moreover, immigration status [26] and Ashkenazi ethnicity [37] in particular, were associated with increased risk for adolescent elevated BP. This explanation is less probable since we analyzed separately Israeli-born adolescents and observed similar trends to that of the entire study population.

Our study lacks data of behavioral factors that are associated with hypertension. While dietary changes are associated with hypertension, multinational long-term cross-sectional studies have indicated stable rates of salt consumption across recent decades [38, 39]. Similarly, fruit and vegetable consumption, which are recommended to reduce blood pressure, remained stable over the last four decades in Israel [40]. Furthermore, previous reports from adolescent populations concluded that salt intake and fruit consumption could not explain BP trends [29] among adolescents. Smoking prevalence among adolescents in Western countries is decreasing [41]; however, its role was shown to be minor regarding SBP and DBP trends among adolescents [29]. Similarly, changes in physical activity [16, 29] or in daily alcohol drinking among adolescents are unlikely to explain the BP trends. Notably, alcohol abuse is more than five-fold less frequent in Israel than in other Western countries such as the US. The effects of other factors on BP are more controversial; for example, the possibility of associations of birth weight and breastfeeding with adult and adolescent hypertension [16, 18, 42].

The results of our study are of public health significance since elevated blood pressure among adolescents plays a substantial role in future cardiovascular morbidity and mortality [3, 43]. This effect is mediated through the increased risk for adulthood hypertension as well as via induction of additional cardiovascular risk factors such as chronic kidney disease and diabetes [4, 5]. Recently, adolescent hypertension has been shown to promote early onset type 2 diabetes [5] which has even more deleterious cardiovascular sequela compared to later onset diabetes [44]. Moreover, elevated blood pressure is a central factor of the metabolic syndrome [45] representing insulin resistance which is induced by obesity [46]. Nevertheless, we observed opposite trends of the BMI and the BP among adolescents throughout the study period, while other traditional environmental and behavioral risk factors are considered to remain stable throughout the years. This suggests that additional factors, that are not traditionally addressed [47], play role in the pathogenesis of elevated blood pressure among adolescents.

This study has limitations. First, throughout the study period the devices used for BP measurements changed. This, however, cannot be the sole explanation for the results as the decreasing trend in DBP started before the digital oscillometric devices were applied and continued after they were routinely  implemented in the medical examination. Second, we lacked lifestyle data. Finally, our study did not include some ethnicities, such as East Asians. Yet, the genetic ancestry of the population was heterogeneous, and was shown to be related by genotyping to contemporary Middle East, North African, European and other Western populations [48, 49]. The strengths of this study include a nationwide screening setting that applied to both sexes, measured BP, weight and height within a narrow age range during a period of over four decades.

In conclusion, we report a trend of a decrement in DBP values during the last four decades in both sexes, which occurred despite significant increases in BMI and in obesity prevalence, but in the presence of more modest changes in SBP.

## Supplementary Information

**Additional file 1: Figure S1.** The secular trends of BMI-adjusted systolic blood pressure and diastolic blood pressure among **A** males (n = 16,27,557) and **B** females (n = 11,57,958), by 5-year time intervals. **Figure S2.** The secular trends of systolic blood pressure, diastolic blood pressure and BMI among **A** males (n= 11,47,611) and **B** females (n = 8,17,085) with unimpaired health, by 5-year time intervals. **Figure S3.** The secular trends of systolic blood pressure, diastolic blood pressure and BMI among **A** males (n = 13,67,901) and **B** females (n = 9,96,922) born in Israel, by 5-year time intervals.

## Data Availability

The databases used in our study were based on Israeli Defense Forces registries and are stored on Israeli Defense Forces computers. These computers are connected solely to the military network. These databases cannot be transferred to other computers or shared on the web, due to Israeli Defense Forces data security restrictions.

## Abbreviations

BP: Blood pressure
SBP: Systolic blood pressure
DBP: Diastolic blood pressure
BMI: Body mass index
