# Supplementary material for: The opposing trends of body mass index and blood pressure during 1977–2020; nationwide registry of 2.8 million male and female adolescents
Source: Cardiovasc Diabetol. 2021 Dec 28;20:242. doi: 10.1186/s12933-021-01433-0 (PMC8715587; doi:10.1186/s12933-021-01433-0)

**The opposing trends of body mass index and blood pressure during 1977-2020; nationwide registry of 2.8 million male and female adolescents**

| Content | Description | page |
| --- | --- | --- |
| Figure S1 | The secular trends of BMI-adjusted systolic blood pressure and diastolic blood pressure | 2 |
| Figure S2 | The secular trends of systolic blood pressure, diastolic blood pressure and BMI among males and females with unimpaired health | 3 |
| Figure S3 | The secular trends of systolic blood pressure, diastolic blood pressure and BMI among males and females born in Israel | 4 |

Figure S1: The secular trends of BMI-adjusted systolic blood pressure and diastolic blood pressure among (A) males (n= 1,627,557) and (B) females (n= 1,157,958), by five-year time intervals.


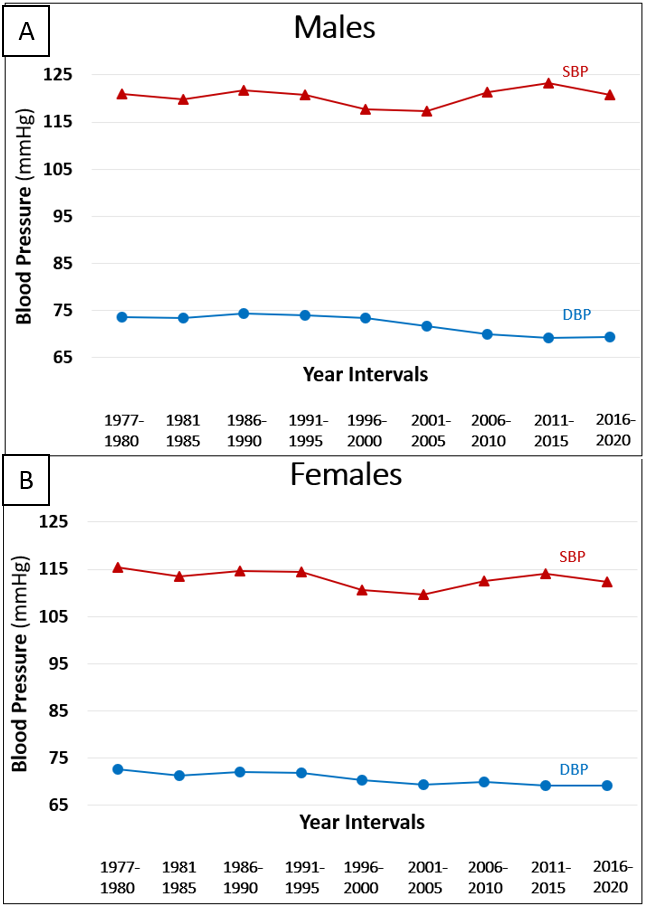


The BMI-adjusted means of systolic blood pressure (SBP) and diastolic blood pressure (DBP) in each time interval were calculated using analysis of covariance (ANCOVA).

Figure S2: The secular trends of systolic blood pressure, diastolic blood pressure and BMI among (A) males (n= 1,147,611) and (B) females (n= 817,085) with unimpaired health, by five-year time intervals.


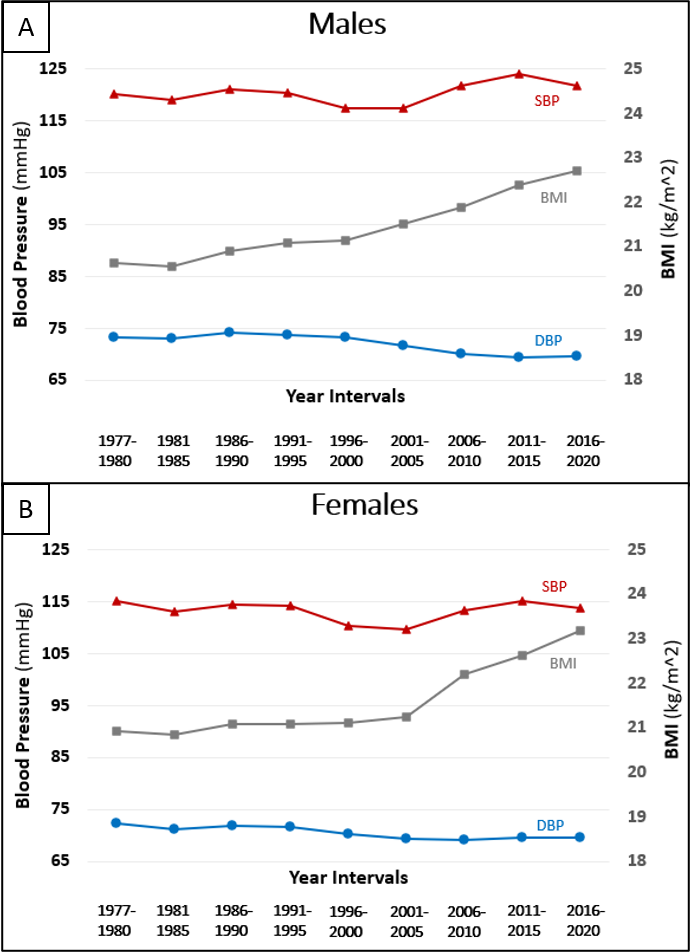


Unimpaired health (apart from hypertension, lack of chronic medical treatment and of a history of major surgery or cancer)

Figure S3: The secular trends of systolic blood pressure, diastolic blood pressure and BMI among (A) males (n= 1,367,901) and (B) females (n= 996,922) born in Israel, by five-year time intervals.


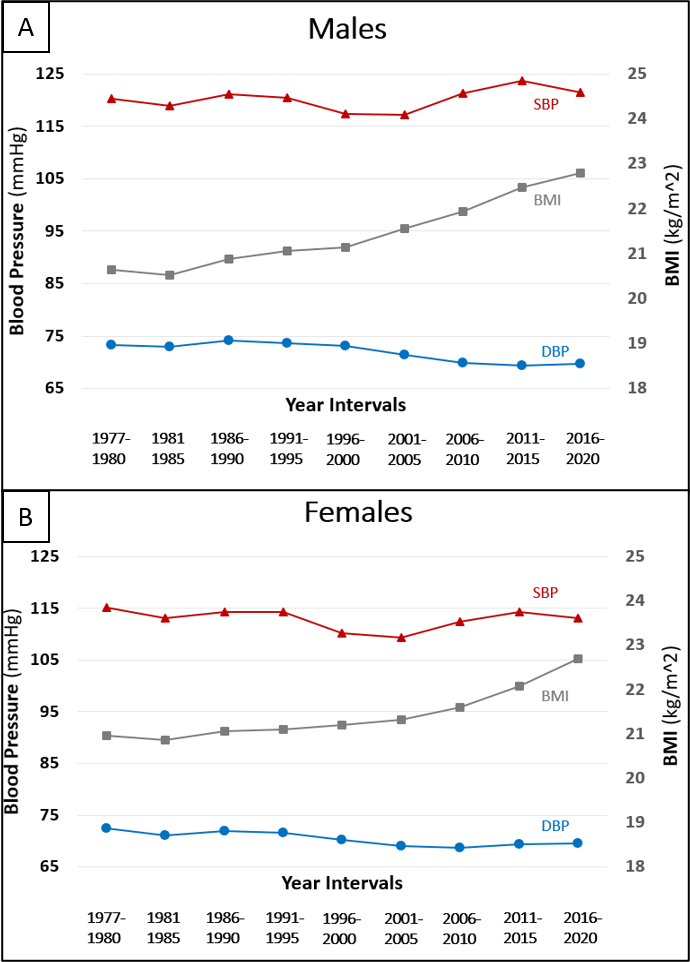

Supplement: Supplementary file 1 — Additional file 1: Figure S1. The secular trends of BMI-adjusted systolic blood pressure and diastolic blood pressure among A males (n = 16,27,557) and B females (n = 11,57,958), by 5-year time intervals. Figure S2. The secular trends of systolic blood pressure, diastolic blood pressure and BMI among A males (n= 11,47,611) and B females (n = 8,17,085) with unimpaired health, by 5-year time intervals. Figure S3. The secular trends of systolic blood pressure, diastolic blood pressure and BMI among A males (n = 13,67,901) and B females (n = 9,96,922) born in Israel, by 5-year time intervals. [file 12933_2021_1433_MOESM1_ESM.docx]
